# The mediation and interaction of body mass index between night shift frequency and hypertension among nursing staff

**DOI:** 10.1186/s12913-026-15399-w

**Published:** 2026-08-20

**Authors:** Ni Chen, Fan Wang, Jie Liu, Jing Li, Suzhi Zhang, Wenjia Liu, Haitao Yang, Bin Zhao

**Affiliations:** 1 Office of Academic Research, The Second Hospital of Hebei Medical University, 215 Heping West Road, Shijiazhuang, Hebei Province China; 2 Division of Health Statistics, School of Public Health, Hebei Medical University, 361 East Zhongshan Road, Shijiazhuang, Hebei Province China; 3 Hebei Key Laboratory of Environment and Human Health, 361 East Zhongshan Road, Shijiazhuang, Hebei Province China; 4 Hebei Key Laboratory of Forensic Medicine, 361 East Zhongshan Road, Shijiazhuang, Hebei Province China; 5 Department of Nursing, The Second Hospital of Hebei Medical University, 215 Heping West Road, Shijiazhuang, Hebei Province China

**Keywords:** Mediation and Interaction Analysis, Body mass index, Night shift frequency, Hypertension prevalence, Nursing staff

## Abstract

**Background:**

Night shift work is associated with obesity and hypertension prevalence among nursing staff, and Body Mass Index (BMI), as an important indicator of obesity, may be involved in this association. This study examined the mediation and interaction components of BMI in the association between night shift frequency and hypertension prevalence. It also explored variations across gender, parental hypertension status, and hospital level.

**Methods:**

A cross-sectional survey conducted from November 2016 to July 2018 included 143,772 nursing staff from 512 hospitals in 11 cities in Hebei Province, China. Using the four-way decomposition method in R software, mediation and interaction components were evaluated, with effect sizes reported as estimates and 95% confidence intervals (95% CI).

**Results:**

Analysis of 127,715 participants showed significant associations between night shift frequency per month (“>0 and ≤ 5”, “>5 and ≤ 10”, “>10”, and “irregular”) and hypertension prevalence [Estimates and 95% CI: 1.09 (1.06, 1.11), 1.18 (1.12, 1.23), 1.28 (1.20, 1.37), and 1.38 (1.27, 1.51), respectively; *P* < 0.001]. Larger controlled direct effect (CDE) estimates were observed among males, whereas significant interaction components between BMI and night shift frequency were identified among females. Nursing staff with parental hypertension and those working in primary-level hospitals showed larger CDE and interaction component estimates, particularly among those with frequent or irregular night shifts.

**Conclusion:**

Higher night shift frequency was significantly associated with higher hypertension prevalence among nursing staff, with direct associations accounting for most of the observed associations. Significant BMI-related mediation and interaction components were mainly observed among nursing staff in primary-level hospitals with frequent or irregular night shifts, suggesting that these BMI-related pathways may be context-dependent.

**Supplementary Information:**

The online version contains supplementary material available at https://doi.org/10.1186/s12913-026-15399-w.

## Introduction

The rising incidence of hypertension has emerged as a significant public health concern on a global scale. According to the Global Hypertension Report 2023 published by the World Health Organization [1], approximately 33% of adults aged 30 to 79 worldwide are affected by hypertension, underscoring the severe prevalence of this condition globally. In China, the prevalence of hypertension among adults within the same age group is reported to be 27%, which translates to approximately 256.7 million individuals. Furthermore, the rates of awareness, treatment, and control of hypertension in China are 52%, 39%, and 16%, respectively, indicating a relatively low overall performance in managing this condition. These statistics highlight the substantial potential for improvement in hypertension management. Consequently, the prevention and management of hypertension represent critical global health issues and pose urgent challenges within China’s public health sector.

With the ongoing evolution of the socio-economic landscape, night shift work has become increasingly prevalent across various industries, particularly within the healthcare sector. Nursing staff, as a critical occupational group, frequently engage in rotating night shifts. Frequent night shift work may disrupt work-life integration by affecting sleep patterns, eating behaviors, psychological recovery, and long-term health management among nursing staff. Previous studies have reported significant associations between night shift work and various health conditions, including hypertension and obesity [2–4]. Notably, female nursing staff working night shifts have shown relatively high rates of obesity [5], suggesting a potential association between night shift work and obesity. As an important indicator of obesity, Body Mass Index (BMI) is strongly associated with hypertension prevalence [6] and may also be involved in the mediation and interaction components of the association between night shift frequency and hypertension prevalence. However, the mediation and interaction components involving BMI in this association have not been fully elucidated. As a result, it remains unclear whether BMI-related changes associated with night shift frequency may be linked to higher hypertension prevalence among nursing staff. This may limit the development of targeted hypertension prevention strategies for nursing staff.

In addition, factors such as gender, family history of hypertension, and hospital level may influence the mediation and interaction components involving BMI in the association between night shift frequency and hypertension prevalence. However, these potential pathways remain unclear, which may further limit the implementation of personalized health management strategies. Therefore, this study aimed to explore the mediation and interaction components involving BMI in the association between night shift frequency and hypertension prevalence, and to provide evidence for the development of personalized intervention strategies for nursing staff.

The association between exposure and outcome can be explored through the concepts of mediation and interaction, which help clarify how a particular factor may be involved in an observed association and in which populations this association may be more pronounced [7]. In the present study, the mediation hypothesis posits that higher night shift frequency among nursing staff may be associated with higher BMI, which may in turn be related to higher hypertension prevalence under specific occupational conditions rather than uniformly across all nursing populations. Conversely, the interaction hypothesis suggests that the association between BMI and hypertension prevalence may be more pronounced among nursing staff who frequently engage in night shifts. Furthermore, mediation and interaction components may coexist. Traditional mediation analysis, however, is often inadequate for distinguishing or elucidating these components. Consequently, this study employed VanderWeele’s four-way decomposition method to analyze mediation and interaction components [8]. This method decomposes the total effect into four distinct components: (1) the effect of exposure in the absence of the mediator, (2) the interaction effect when the mediator is held constant at its pre-exposure level, (3) the mediated interaction effect (INT_med_), and (4) the pure indirect effect (PIE). The four-way decomposition method facilitates the simultaneous evaluation of mediation and interaction components, thereby providing a comprehensive analysis of complex exposure–mediator–outcome associations. By decomposing the total effect into these four components, this method allows for quantification of each component and enhances the understanding of the relationships among exposure, outcome, and mediator. This approach may provide useful evidence for the development of targeted interventions [9, 10].

This study employed the four-way decomposition method to investigate the mediation and interaction components involving BMI in the association between night shift frequency and hypertension prevalence. The findings may provide evidence to support the prevention and management of hypertension among nursing staff.

## Methods

### Study population

In this cross-sectional study, we utilized a census approach to survey all registered nurses from 512 medical institutions across 11 cities in Hebei Province, China. The questionnaire was developed for this study, and further details are provided in the Supplementary Material. The inclusion criteria were as follows: (1) Registered nurses with at least one year of nursing experience who are currently employed in a nursing position; (2) Individuals aged 18 years or older; (3) Participants with clear mental status, free from psychiatric disorders; (4) Provision of informed consent and voluntary participation. The exclusion criteria included nurses who were on sick leave, maternity leave, or engaged in external training programs. This study received approval from the Ethics Committee of the Second Hospital of Hebei Medical University (Approval Number: 2016225).

**Fig. 1:**
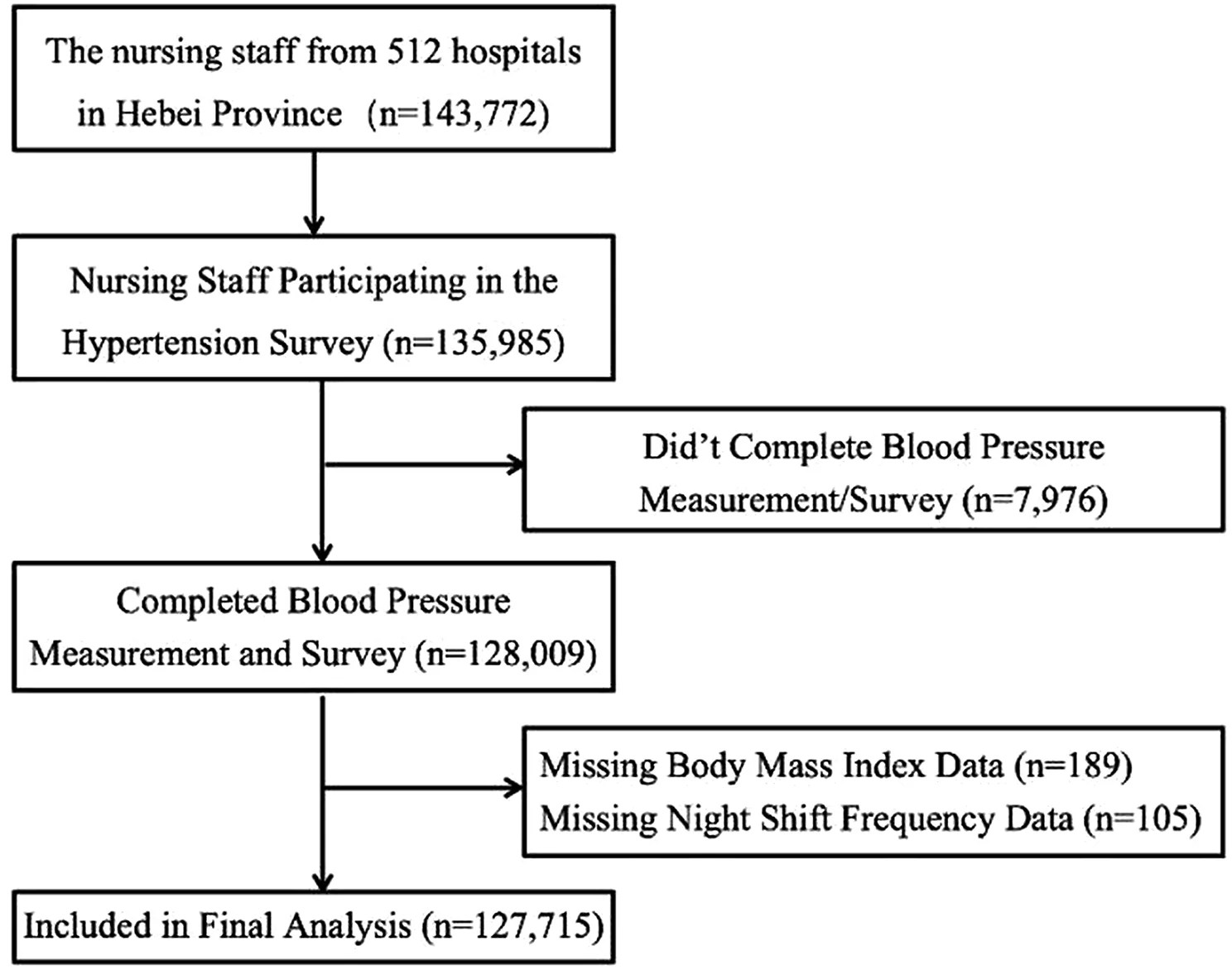
Flowchart of participant selection

As illustrated in the flow chart (Fig. 1. Flowchart of Participant Selection), a total of 135,985 out of 143,772 nursing staff members who were part of this study completed the survey. A total of 128,009 valid questionnaires were obtained. Following the exclusion of 189 cases with incomplete Body Mass Index (BMI) data and 105 cases with missing information regarding night shift frequency over the past six months, the final analysis included 127,715 participants.

### Assessment of night shift frequency

The frequency of night shifts over the preceding six months was evaluated through the administration of a questionnaire. The specific inquiry posed was: “In the past six months, how many night shifts did you work on average per month?” The available response options included: “0 shifts,” “>0 and ≤ 5 shifts,” “>5 and ≤ 10 shifts,” “>10 shifts,” and “Irregular.”

### Assessment of the mediator variable BMI

Height and weight data of the nursing staff were self-reported through questionnaires based on the results of their most recent physical examinations. BMI was calculated as weight/height² (kg/m²), and participants were categorized according to the following criteria: <28.0 kg/m² as non-obese and ≥ 28.0 kg/m² as obese. The BMI cutoff was based on the Chinese obesity classification standard. In the four-way decomposition analysis, dichotomization of BMI facilitated interpretation of the controlled direct effect under a clinically meaningful obesity threshold.

### Assessment of hypertension

The blood pressure measurement tools utilized in this study included upper-arm electronic medical blood pressure monitors, which were regularly calibrated in each department and certified according to international standards, as well as mercury sphygmomanometers that adhered to established measurement criteria. Blood pressure was measured by nursing staff in each department following a standardized measurement protocol issued by the Hebei Provincial Nursing Quality Control Center to ensure consistency across the measurement tools. A standard cuff (22–26 cm in length and 12 cm in width) was used for most participants, while a larger cuff was employed for individuals with an upper arm circumference > 32 cm, ensuring that the cuff bladder covered at least 80% of the upper arm. Participants were instructed to refrain from smoking, and consuming coffee or tea for at least 30 min before the measurement, to empty their bladder, and to rest quietly in a seated position for a minimum of 5 min. Measurements were taken with the participant seated, with their back supported, upper arm exposed, and positioned at heart level. Blood pressure was measured on the right upper arm, with three consecutive readings taken at 1–2 min intervals. The second and third readings were averaged, while the first reading was discarded to reduce the influence of initial measurement variability.

The diagnostic criteria for hypertension were based on the Chinese Guidelines for the Prevention and Treatment of Hypertension (2018 Revision) [11]. In adults, hypertension was defined as systolic blood pressure (SBP) ≥ 140 mmHg (1 mmHg = 0.133 kPa) and/or diastolic blood pressure (DBP) ≥ 90 mmHg in the absence of antihypertensive medication. Individuals with a prior history of hypertension who were currently taking antihypertensive medication but maintained a blood pressure of < 140/90 mmHg were still classified as hypertensive.

### Assessment of covariates

The questionnaire also gathered information on participants’ gender, age, smoking habits, alcohol consumption, exercise activity, parental history of hypertension, and the level of the hospital where they were employed.

1. Smoking: “None” indicates that there has been no smoking behavior in the past year. “<10 cigarettes/day,” “10–20 cigarettes/day,” and “>20 cigarettes/day” indicate the average daily smoking amount.
2. Alcohol Consumption: “None” indicates no alcohol consumption in the past year; “Occasional” refers to drinking less than once per month on average during the past year; “Frequent” denotes drinking at least once per month on average over the past year.
3. Exercise Activity: This term refers to activities such as running or walking that are performed outside of work and consciously engaged in for more than 30 min. “Never” denotes no regular participation in such activities over the past year. “Occasional” denotes participation in such activities 1 to 2 times per week on average over the past year, while “Frequent” denotes participation at least 3 times per week on average during the same period.
4. Parental Hypertension: At least one parent has been diagnosed with hypertension.
5. Hospital Level: The hospital level refers to the classification of the facility where the participant is employed, which can be categorized as a “Primary-level hospital,” “Secondary hospital,” or “Tertiary hospital.”

### Statistical analysis

**Fig. 2:**
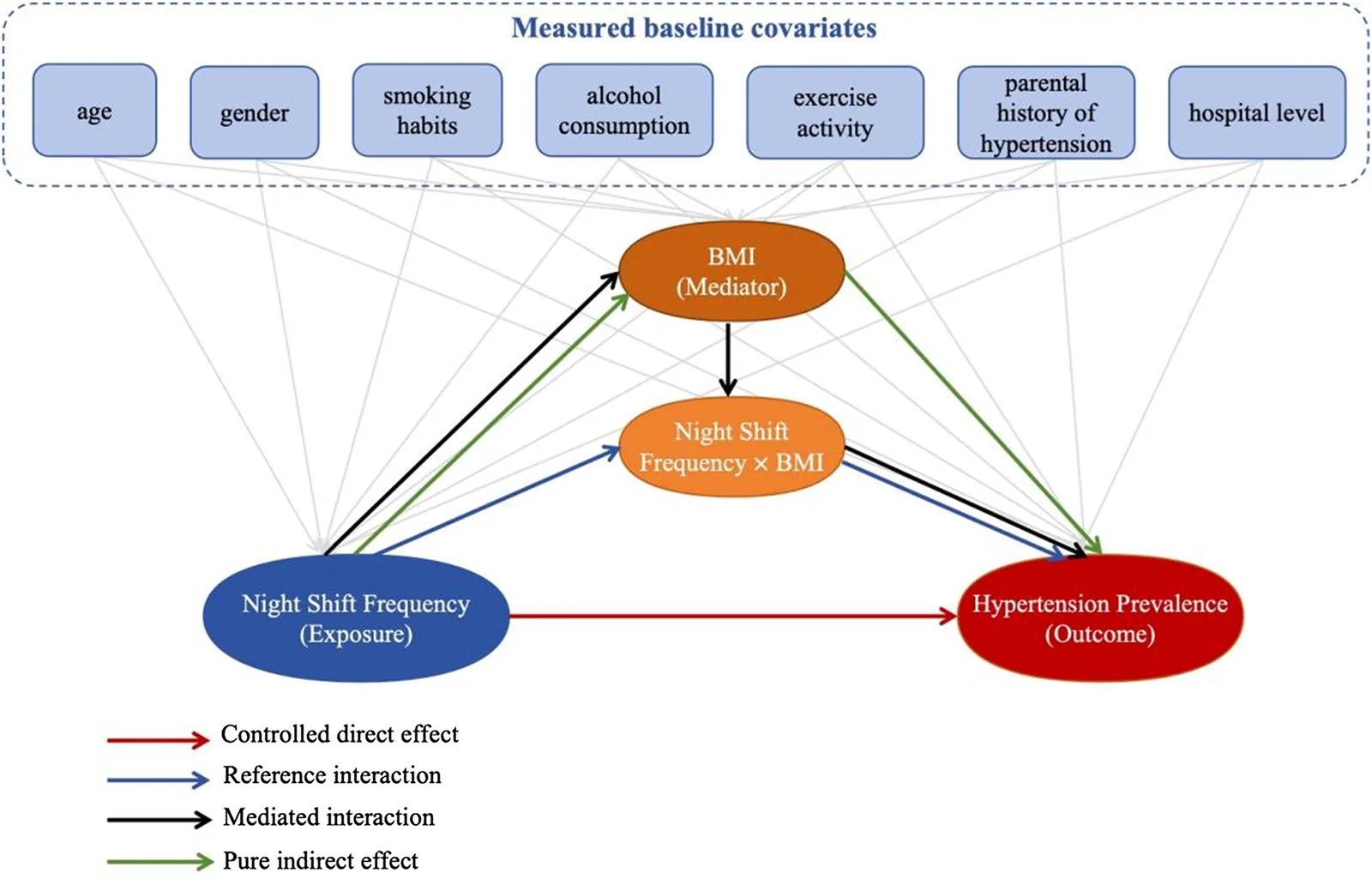
Conceptual directed acyclic graph (DAG) illustrating the assumed relationships among night shift frequency (exposure), BMI (mediator), hypertension prevalence (outcome), and the measured baseline covariates adjusted for in the four-way decomposition analysis

The four-way decomposition method developed by J. VanderWeele was employed to analyze the mediation and interaction components involving BMI in the association between night shift frequency and hypertension prevalence among nursing staff. Given the cross-sectional design of this study, the decomposition results were interpreted as components of observed associations rather than definitive causal effects or causal pathways. This analysis was conducted overall and stratified by gender, parental hypertension status, and hospital level. The four-way decomposition analysis was conducted on the additive scale. A conceptual directed acyclic graph (DAG) illustrating the assumed relationships among night shift frequency, BMI, hypertension prevalence, and the measured baseline covariates is presented in Fig. 2. The DAG provides the conceptual framework underlying the four-way decomposition analysis. The mediation and interaction analysis decomposed the total effect into four components: controlled direct effect (CDE), reference interaction (INT_ref_), mediated interaction (INT_med_), and pure indirect effect (PIE). The specific definitions of these components in this study are as follows:

1. CDE: The controlled direct effect component representing the association between night shift frequency and hypertension prevalence when BMI was controlled.
2. INT_ref_: The reference interaction component representing the interaction between night shift frequency and obesity unrelated to night shifts in relation to hypertension prevalence.
3. INT_med_: The mediated interaction component representing the combined mediation and interaction component between night shift frequency and BMI in relation to hypertension prevalence.
4. PIE: The pure indirect effect component representing the BMI-related indirect association between night shift frequency and hypertension prevalence.

**Fig. 3:**
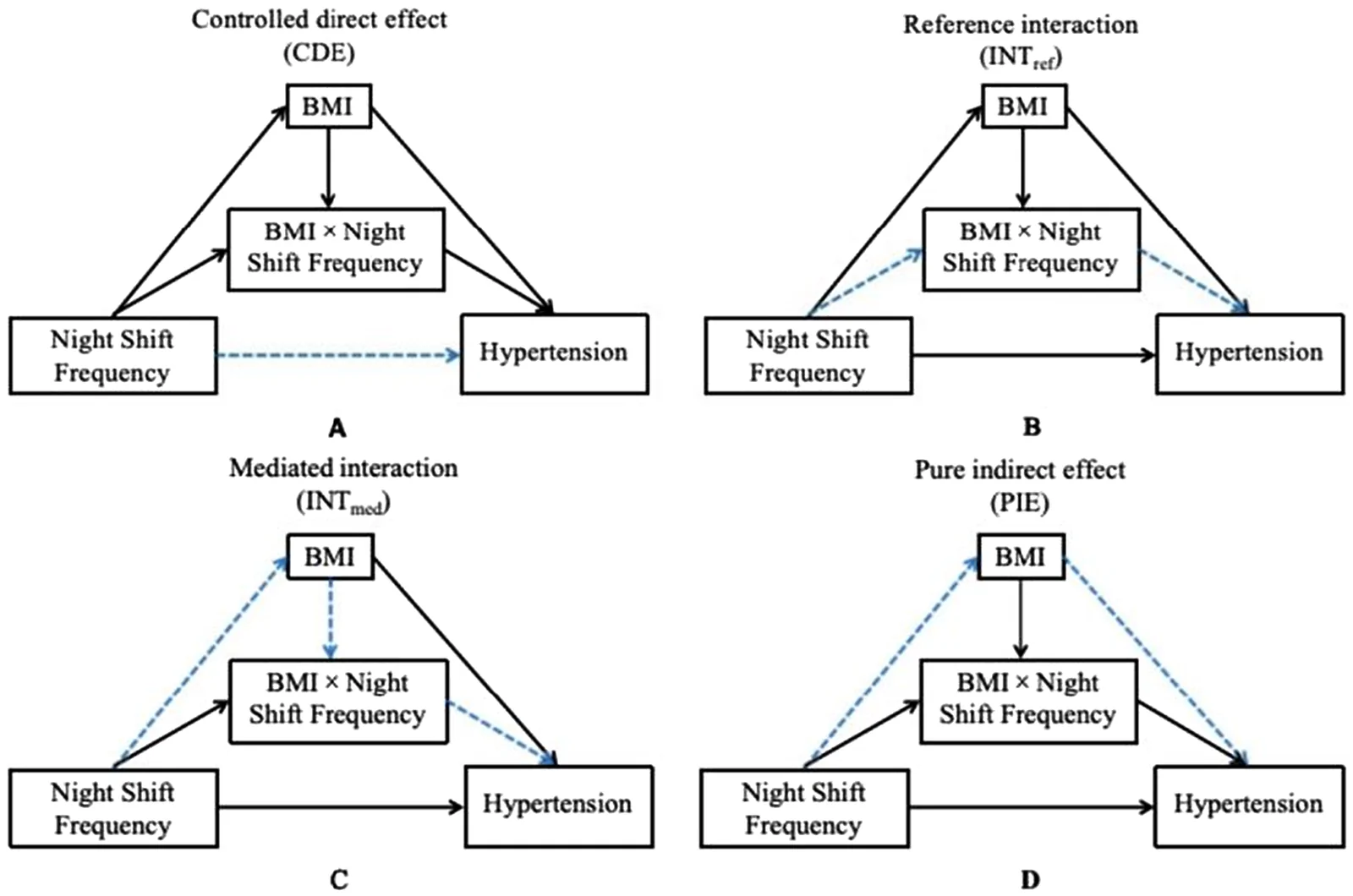
Four-Way Decomposition Diagram in Mediation and Interaction Analysis. Note: Blue arrows represent the four decomposed components; (**A**) CDE refers to the controlled direct effect component, representing the association between night shift frequency and hypertension prevalence when BMI was controlled. (**B**) INT_ref_ refers to the reference interaction component, representing the interaction between night shift frequency and obesity, conditional to no night shifts in relation to hypertension prevalence. (**C**) INT_med_ refers to the mediated interaction component, representing the combined mediation and interaction component between night shift frequency and BMI in relation to hypertension prevalence. (**D**) PIE refers to the pure indirect effect component, representing the BMI-related indirect association between night shift frequency and hypertension prevalence

Figure 3 (Four-Way Decomposition Diagram in Mediation and Interaction Analysis) illustrates the association between night shift frequency and hypertension risk, as well as the potential mediating and interaction roles of BMI. The analysis utilized the “CMAverse” package in R, employing regression-based methods and counterfactual imputation. Logistic regression models were applied to examine the associations between night shift frequency and BMI, as well as between night shift frequency and hypertension prevalence, while controlling for potential confounding factors including age, smoking, alcohol consumption, and physical activity. Nurses without night shifts were used as the reference group for comparisons across different night shift frequency categories. Standard errors and P-values for the effect estimates were calculated using Bootstrap with 1500 iterations. Excess relative risk (ERR) and 95% confidence intervals (95% CI) were also computed. ERR was calculated as total effect odds ratio(Rte) minus 1, under the counterfactual framework, the odds ratio comparing the occurrence of the outcome event between two exposure levels [12]. As a sensitivity analysis, the four-way decomposition analyses were repeated with BMI modeled as a continuous mediator to evaluate the robustness of the primary findings. The same exposure categories, outcome definition, covariate adjustment, estimation procedure, and Bootstrap settings were retained as in the primary analyses. A P-value of less than 0.05 was considered statistically significant.

## Results

### Four-way decomposition of the overall association between night shift frequency and hypertension among nursing staff, with BMI as a potential mediator

As shown in Table 1, compared with nurses who did not work night shifts, the total effect estimates indicated significant associations between night shift frequency and hypertension prevalence among nursing staff across different night shift frequency groups (*P* < 0.05). The estimates increased with higher night shift frequency, with the strongest association observed in the irregular night shift group.

When BMI was controlled at 28 kg/m² or below, statistically significant CDE components were observed across all hospital levels and night shift frequency groups (*P* < 0.05), accounting for the largest proportion of the total effect in all groups, with all proportions exceeding 70%.

The INT_ref_ component between non-shift-related obesity and night shift frequency was statistically significant across all night shift frequency groups (*P* < 0.05).

**Table 1:** Overall four-way decomposition of the association between night shift frequency and hypertension prevalence among nursing staff

Night Shift Frequency Grouping in the Past Six Months	Comparison of BMI ≤ 28 and > 28 [Estimate (95% CI)]			
	> 0 and ≤ 5	>5 and ≤ 10	>10	Irregular
Total effect	1.09(1.07,1.12)*	1.19(1.14,1.24)*	1.30(1.21,1.38)*	1.42(1.29,1.54)*
ERR due to each component				
CDE	0.07(0.05,0.09)*	0.14(0.10,0.19)*	0.22(0.15,0.30)*	0.31(0.20,0.42)*
INT_ref_	0.02(0.01,0.03)*	0.05(0.03,0.06)*	0.07(0.04,0.10)*	0.11(0.06,0.15)*
INT_med_	-0.00(-0.00,0.00)	0.00(-0.00,0.00)	0.00(-0.00,0.01)	0.00(-0.01,0.01)
PIE	-0.00(-0.00,0.01)	-0.00(-0.01,0.01)	0.00(-0.01,0.01)	0.00(-0.01,0.01)
Proportion of effect due to each component				
CDE	0.76(0.65,0.86)*	0.76 (0.65, 0.85)*	0.75 (0.64, 0.85)*	0.74 (0.63, 0.84)*
INT_ref_	0.24(0.15,0.34)*	0.24 (0.16, 0.35)*	0.25 (0.16, 0.36)*	0.26 (0.17, 0.37)*
INT_med_	-0.00(-0.01,0.01)	0.00 (-0.01, 0.01)	0.00 (-0.02, 0.02)	0.00 (-0.02, 0.02)
PIE	-0.00(-0.06,0.05)	-0.00 (-0.04, 0.04)	0.00 (-0.03, 0.03)	0.00 (-0.03, 0.03)

Note: *P<0.05. BMI:Body Mass Index; CDE:controlled direct effect; INT_ref_:reference interaction; INT_med_:mediated interaction; PIE:pure indirect effect

### Four-way decomposition of the association between night shift frequency and hypertension among nursing staff of different genders, with BMI as a potential mediator

As shown in Table 2, compared with nurses who did not work night shifts, the total effect estimates indicated significant associations between night shift frequency and hypertension prevalence in both female and male nursing staff across different night shift frequency groups (*P* < 0.05). The estimates increased with higher night shift frequency, with the strongest associations observed in the irregular night shift group, particularly among males.

When BMI was controlled at 28 kg/m² or below, statistically significant CDE components were observed in both female and male nursing staff across all night shift frequency groups (*P* < 0.05). The CDE accounted for the largest proportion of the total effect in both genders, with all proportions exceeding 70%.

The INT_ref_ component between non-shift-related obesity and night shift frequency was statistically significant among female nursing staff across all night shift frequency groups (*P* < 0.05), whereas the corresponding INT_ref_ components were not statistically significant among male nursing staff (*P* > 0.05).

**Table 2:** Four-way decomposition of the effect of night shift frequency on hypertension among nursing staff of different genders, with bmi as a potential mediator

Night Shift Frequency Grouping in the Past Six Months	Group	Comparison of BMI ≤ 28 and > 28 [Estimate (95% CI)]					
		> 0 and ≤ 5	>5 and ≤ 10			>10	Irregular
Total effect	Female	1.06 (1.03, 1.09)*	1.13 (1.07, 1.18)*			1.19 (1.11, 1.28)*	1.26 (1.14, 1.39)*
	Male	1.10 (1.03, 1.19)*	1.22 (1.06, 1.41)*			1.35 (1.09, 1.67)*	1.49 (1.12, 1.98)*
Total effect ERR due to each component							
CDE	Female	0.05 (0.02, 0.07)*	0.10 (0.05, 0.15)*			0.15 (0.07, 0.23)*	0.20 (0.09, 0.32)*
	Male	0.08 (0.02, 0.14)*	0.16 (0.04, 0.31)*			0.25 (0.06, 0.49)*	0.35 (0.09, 0.69)*
INT_ref_	Female	0.02 (0.01, 0.02)*	0.03 (0.01, 0.05)*			0.05 (0.02, 0.08)*	0.07 (0.03, 0.12)*
	Male	0.02 (-0.01, 0.05)	0.04 (-0.02, 0.12)			0.07 (-0.04, 0.20)	0.11 (-0.04, 0.31)
INT_med_	Female	0.00 (-0.00, 0.00)	-0.00 (-0.00, 0.00)			-0.00 (-0.01, 0.00)	-0.00 (-0.01, 0.00)
	Male	-0.00 (-0.00, 0.00)	0.00 (-0.01, 0.01)			0.00 (-0.03, 0.03)	0.00 (-0.05, 0.07)
PIE	Female	0.00 (-0.01, 0.00)	-0.00 (-0.01, 0.00)			-0.00 (-0.01, 0.00)	-0.01 (-0.02, 0.00)
	Male	-0.00 (-0.01, 0.03)	0.01 (-0.01, 0.05)			0.02 (-0.01, 0.08)	0.03 (-0.01, 0.10)
Proportion of effect due to each component							
CDE	Female	0.74 (0.59, 0.94)*	0.76 (0.59, 0.92)*			0.76 (0.58, 0.91)*	0.76 (0.58, 0.91)*
	Male	0.82 (0.41, 1.15)*	0.75 (0.39, 1.10)*			0.73 (0.37, 1.11)*	0.71 (0.35, 1.12)*
INT_ref_	Female	0.25 (0.13, 0.46)*	0.26 (0.13, 0.48)*			0.27 (0.14, 0.49)*	0.27 (0.14, 0.50)*
	Male	0.20 (-0.26, 0.47)	0.19 (-0.23, 0.46)			0.20 (-0.21, 0.47)	0.22 (-0.20, 0.47)
INT_med_	Female	0.00 (-0.02, 0.00)	-0.00 (-0.03, 0.00)			-0.01 (-0.04, 0.00)	-0.01 (-0.05, 0.01)
	Male	-0.00 (-0.04, 0.03)	0.00 (-0.08, 0.06)			0.00 (-0.12, 0.09)	0.01 (-0.18, 0.12)
PIE	Female	0.01 (-0.15, 0.04)	-0.01 (-0.12, 0.02)			-0.02 (-0.10, 0.01)	-0.02 (-0.10, 0.01)
	Male	-0.01 (-0.10, 0.36)	0.06 (-0.03, 0.34)			0.06 (-0.02, 0.34)	0.07 (-0.02, 0.35)

Note: *P < 0.05. BMI: Body Mass Index; CDE: controlled direct effect; INT_ref_:reference interaction; INT_med_:mediated interaction; PIE: pure indirect effect

### Four-way decomposition of the association between night shift frequency and hypertension among nursing staff with and without parental hypertension, with BMI as a potential mediator

As shown in Table 3, compared with nurses who did not work night shifts, the total effect estimates indicated significant associations between night shift frequency and hypertension prevalence among nursing staff both with and without parental hypertension across different night shift frequency groups (*P* < 0.05). The estimates increased with higher night shift frequency, with the strongest associations observed in the irregular night shift group. In addition, the estimates were consistently larger among nursing staff with parental hypertension than among those without parental hypertension.

When BMI was controlled at 28 kg/m² or below, statistically significant CDE components were observed among nursing staff both with and without parental hypertension across all night shift frequency groups (*P* < 0.05). The CDE accounted for the largest proportion of the total effect in both groups, with all proportions exceeding 70%.

The INT_ref_ component between non-shift-related obesity and night shift frequency was statistically significant among nursing staff with parental hypertension across all night shift frequency groups (*P* < 0.05). Corresponding INT_ref_ components were also statistically significant among nursing staff without parental hypertension, although the estimates were relatively smaller (*P* < 0.05).

**Table 3:** Four-way decomposition of the effect of night shift frequency on hypertension among nursing staff with and without parental hypertension, with bmi as a potential mediator

Night Shift Frequency Grouping in the Past Six Months	Group	Comparison of BMI ≤ 28 and > 28 [Estimate (95% CI)]			
		> 0 and ≤ 5	>5 and ≤ 10	>10	Irregular
Total effect	Parents with Hypertension	1.11 (1.08, 1.15)*	1.23 (1.16, 1.31)*	1.37 (1.25, 1.50)*	1.52 (1.34, 1.72)*
	Parents without Hypertension	1.07 (1.04, 1.11)*	1.14 (1.07, 1.23)*	1.22 (1.11, 1.36)*	1.31 (1.15, 1.50)*
Total effect ERR due to each component					
CDE	Parents with Hypertension	0.08 (0.05, 0.11)*	0.17 (0.11, 0.24)*	0.27 (0.17, 0.38)*	0.37 (0.23, 0.54)*
	Parents without Hypertension	0.06 (0.02, 0.09)*	0.12 (0.05, 0.19)*	0.18 (0.07, 0.30)*	0.24 (0.10, 0.41)*
INT_ref_	Parents with Hypertension	0.03 (0.02, 0.04)*	0.06 (0.04, 0.09)*	0.10 (0.06, 0.15)*	0.15 (0.09, 0.22)*
	Parents without Hypertension	0.01 (0.00, 0.02)*	0.03 (0.00, 0.05)*	0.04 (0.00, 0.09)*	0.06 (0.01, 0.12)*
INT_med_	Parents with Hypertension	-0.00 (-0.00, 0.00)	-0.00 (-0.00, 0.00)	-0.00 (-0.01, 0.01)	-0.00 (-0.02, 0.01)
	Parents without Hypertension	-0.00 (-0.00, 0.00)	-0.00 (-0.00, 0.00)	0.00 (-0.00, 0.01)	0.00 (-0.01, 0.01)
PIE	Parents with Hypertension	-0.00 (-0.01, 0.01)	-0.00 (-0.01, 0.01)	-0.00 (-0.01, 0.01)	-0.00 (-0.02, 0.01)
	Parents without Hypertension	-0.00 (-0.01, 0.01)	-0.00 (-0.01, 0.01)	0.00 (-0.01, 0.02)	0.00 (-0.01, 0.02)
Proportion of effect due to each component					
CDE	Parents with Hypertension	0.75 (0.62, 0.86)*	0.74 (0.61, 0.84)*	0.73 (0.61, 0.83)*	0.72 (0.59, 0.83)*
	Parents without Hypertension	0.83 (0.56, 1.00)*	0.80 (0.56, 0.98)*	0.79 (0.55, 0.98)*	0.79 (0.54, 0.97)*
INT_ref_	Parents with Hypertension	0.27 (0.17, 0.38)*	0.27 (0.18, 0.39)*	0.28 (0.18, 0.40)*	0.29 (0.19, 0.41)*
	Parents without Hypertension	0.20 (0.01, 0.42)*	0.20 (0.02, 0.42)*	0.20 (0.02, 0.43)*	0.20 (0.03, 0.44)*
INT_med_	Parents with Hypertension	-0.00 (-0.01, 0.01)	-0.00 (-0.02, 0.01)	-0.00 (-0.03, 0.02)	-0.01 (-0.04, 0.03)
	Parents without Hypertension	-0.00 (-0.01, 0.01)	-0.00 (-0.01, 0.02)	0.00 (-0.02, 0.03)	0.00 (-0.02, 0.04)
PIE	Parents with Hypertension	-0.02 (-0.08, 0.06)	-0.01 (-0.06, 0.04)	-0.01 (-0.05, 0.03)	-0.01 (-0.04, 0.03)
	Parents without Hypertension	-0.02 (-0.11, 0.12)	-0.00 (-0.06, 0.09)	0.00 (-0.05, 0.08)	0.01 (-0.05, 0.08)

Note: *P<0.05. BMI:Body Mass Index; CDE:controlled direct effect; INT_ref_:reference interaction; INT_med_:mediated interaction; PIE:pure indirect effect

### Four-way decomposition of the association between night shift frequency and hypertension among nursing staff in hospitals of different levels, with BMI as a potential mediator

As shown in Table 4, compared with nurses who did not work night shifts, the total effect estimates indicated significant associations between night shift frequency and hypertension prevalence among nursing staff across different hospital levels (*P* < 0.05). The estimates increased with higher night shift frequency, with the strongest associations observed in the irregular night shift group. In addition, the estimates were consistently largest among nursing staff in primary-level hospitals.

When BMI was controlled at 28 kg/m² or below, statistically significant CDE components were observed across all hospital levels and night shift frequency groups (*P* < 0.05). The CDE accounted for the largest proportion of the total effect in all groups, with all proportions exceeding 60%.

The INT_ref_ component between non-shift-related obesity and night shift frequency was statistically significant across all hospital levels and night shift frequency groups (*P* < 0.05). In addition, statistically significant INT_med_ and PIE components were observed only among nursing staff in primary-level hospitals with night shift frequencies of “>5 and ≤ 10,” “>10,” and “irregular” (*P* < 0.05).

**Table 4:** Four-way decomposition of the effect of night shift frequency on hypertension among nursing staff in hospitals of different levels, with bmi as a potential mediator

Night Shift Frequency Grouping in the Past Six Months	Group	Comparison of BMI ≤ 28 and > 28 [Estimate (95% CI)]			
		> 0 and ≤ 5	>5 and ≤ 10	>10	Irregular
Total effect	Primary-level	1.26 (1.16, 1.39)*	1.61 (1.36, 1.94)*	2.06 (1.59, 2.71)*	2.65 (1.89, 3.80)*
	Secondary	1.09 (1.05, 1.12)*	1.18 (1.10, 1.26)*	1.28 (1.16, 1.41)*	1.39 (1.22, 1.57)*
	Tertiary	1.07 (1.04, 1.11)*	1.15 (1.07, 1.24)*	1.24 (1.11, 1.38)*	1.33 (1.15, 1.53)*
Total effect ERR due to each component					
CDE	Primary-level	0.20 (0.10, 0.30)*	0.43 (0.20, 0.69)*	0.70 (0.31, 1.19)*	1.02 (0.43, 1.80)*
	Secondary	0.07 (0.04, 0.10)*	0.14 (0.08, 0.21)*	0.22 (0.12, 0.33)*	0.30 (0.16, 0.46)*
	Tertiary	0.06 (0.02, 0.09)*	0.11 (0.04, 0.19)*	0.18 (0.06, 0.30)*	0.24 (0.07, 0.42)*
INT_ref_	Primary-level	0.05 (0.02, 0.08)*	0.13 (0.05, 0.21)*	0.23 (0.10, 0.39)*	0.37 (0.15, 0.65)*
	Secondary	0.02 (0.01, 0.03)*	0.04 (0.01, 0.06)*	0.06 (0.02, 0.10)*	0.09 (0.03, 0.15)*
	Tertiary	0.02 (0.01, 0.03)*	0.05 (0.02, 0.08)*	0.08 (0.04, 0.12)*	0.11 (0.05, 0.18)*
INT_med_	Primary-level	0.00 (-0.00, 0.02)	0.03 (0.01, 0.08)*	0.08 (0.02, 0.24)*	0.20 (0.04, 0.57)*
	Secondary	0.00 (-0.00, 0.00)	0.00 (-0.00, 0.00)	-0.00 (-0.01, 0.01)	-0.00 (-0.01, 0.01)
	Tertiary	-0.00 (-0.00, 0.00)	-0.00 (-0.01, 0.00)	-0.00 (-0.01, 0.00)	-0.01 (-0.02, 0.01)
PIE	Primary-level	0.01 (-0.00, 0.04)	0.03 (0.01, 0.07)*	0.04 (0.02, 0.11)*	0.06 (0.03, 0.16)*
	Secondary	0.00 (-0.01, 0.01)	0.00 (-0.01, 0.01)	0.00 (-0.02, 0.01)	0.00 (-0.02, 0.02)
	Tertiary	-0.00 (-0.01, 0.00)	-0.01 (-0.01, 0.00)	-0.01 (-0.02, 0.01)	-0.01 (-0.02, 0.01)
Proportion of effect due to each component					
CDE	Primary-level	0.75 (0.56, 0.84)*	0.70 (0.51, 0.82)*	0.66 (0.45, 0.80)*	0.62 (0.39, 0.77)*
	Secondary	0.78 (0.63, 0.96)*	0.78 (0.63, 0.95)*	0.78 (0.62, 0.94)*	0.78 (0.62, 0.93)*
	Tertiary	0.76 (0.45, 0.92)*	0.74 (0.45, 0.90)*	0.74 (0.45, 0.90)*	0.73 (0.45, 0.90)*
INT_ref_	Primary-level	0.21 (0.09, 0.33)*	0.21 (0.10, 0.34)*	0.22 (0.11, 0.34)*	0.22 (0.12, 0.33)*
	Secondary	0.20 (0.07, 0.38)*	0.21 (0.08, 0.38)*	0.22 (0.08, 0.38)*	0.22 (0.09, 0.39)*
	Tertiary	0.31 (0.14, 0.61)*	0.31 (0.15, 0.63)*	0.32 (0.15, 0.65)*	0.33 (0.15, 0.67)*
INT_med_	Primary-level	0.02 (-0.00, 0.07)	0.05 (0.01, 0.13)*	0.08 (0.02, 0.21)*	0.12 (0.03, 0.29)*
	Secondary	0.00 (-0.01, 0.01)	0.00 (-0.02, 0.01)	-0.00 (-0.02, 0.02)	-0.00 (-0.03, 0.02)
	Tertiary	-0.01 (-0.03, 0.01)	-0.01 (-0.05, 0.01)	-0.02 (-0.07, 0.01)	-0.02 (-0.10, 0.02)
PIE	Primary-level	0.02 (-0.01, 0.15)	0.04 (0.01, 0.13)*	0.04 (0.01, 0.12)*	0.04 (0.01, 0.12)*
	Secondary	0.02 (-0.11, 0.08)	0.01 (-0.07, 0.05)	0.00 (-0.06, 0.05)	0.00 (-0.05, 0.04)
	Tertiary	-0.06 (-0.18, 0.06)	-0.04 (-0.12, 0.03)	-0.04 (-0.11, 0.03)	-0.03 (-0.10, 0.02)

Note: *P<0.05. BMI:Body Mass Index; CDE:controlled direct effect; INT_ref_:reference interaction; INT_med_:mediated interaction; PIE:pure indirect effect

### Sensitivity analysis of the four-way decomposition of the association between night shift frequency and hypertension prevalence among nursing staff with BMI modeled as a continuous mediator

Sensitivity analyses modeling BMI as a continuous mediator yielded results generally consistent with the primary analyses using dichotomized BMI. The controlled direct effect remained the predominant component in most analyses, whereas BMI-related mediated components were generally small and were mainly observed in specific subgroups, particularly among nursing staff in primary-level hospitals. Overall, the main interpretation of the findings was not materially altered. Detailed results are presented in Supplementary Tables S1–S4.

## Discussion

### Analysis of the independent and interaction components of night shift frequency associated with hypertension prevalence among nursing staff

This study found that higher night shift frequency was significantly associated with higher hypertension prevalence among nursing staff, with the strongest association observed among those with irregular night shifts. The strongest association observed in the irregular night shift group may suggest that unstable or unpredictable scheduling patterns are more likely to disrupt circadian rhythms and sleep recovery among nursing staff [13]. After controlling for BMI, this association remained statistically significant, which is consistent with previous studies [14, 15]. Additionally, a significant interaction component between obesity and night shift frequency was identified and was associated with higher hypertension prevalence. However, this interaction has been less commonly addressed in previous research. Pan [16] reported a positive association between night shift work, obesity, and type 2 diabetes, and previous evidence also suggests that the association between BMI and hypertension may be stronger among overweight and obese individuals [17]. Several factors may help explain these findings. First, disrupted sleep-wake cycles caused by forced sleep deprivation and exposure to light during night shifts may contribute to metabolic dysregulation associated with cardiovascular health [18]. Second, nurses working night shifts often have irregular eating patterns, including higher caffeine and sugar intake, which may adversely affect metabolism [19]. Smoking among night shift nurses may also be associated with higher hypertension prevalence [20]. Third, occupational stress, which is common during night shift work, may contribute to higher hypertension prevalence [21]. These findings suggest potential health risks associated with irregular night shift scheduling and suggest that more stable and predictable scheduling strategies may help reduce circadian disruption and occupational health burdens among nursing staff. Therefore, future longitudinal studies should further explore these mechanisms and include additional confounders to better clarify these potential pathways.

### Analysis of the CDE components of night shift frequency on hypertension prevalence across genders, parental hypertension status, and hospital levels

This study found that, after controlling for BMI at ≤ 28 kg/m², higher night shift frequency was significantly associated with higher hypertension prevalence among nursing staff, particularly among males. Circadian rhythm disruption, metabolic abnormalities, and psychological stress related to night shift work may contribute to elevated blood pressure among nursing staff [22]. Gender-specific physiological responses may also contribute to these associations [23]. Females may be more sensitive to stress due to hormonal fluctuations, whereas males may experience more pronounced associations between night shift work and hypertension prevalence [24]. The study also indicated that the association between night shift frequency and hypertension prevalence remained statistically significant regardless of parental hypertension history, with larger estimates observed among nursing staff with hypertensive parents, suggesting a potential role of genetic susceptibility. Even among non-obese individuals, night shifts were associated with higher hypertension prevalence [25]. In addition, the association between night shift frequency and hypertension prevalence differed across hospital levels and appeared to be strongest in primary-level hospitals. This finding may be related to relatively limited medical resources, heavier workloads, and increased occupational stress and fatigue among nursing staff in these settings. Notably, in most categories, the CDE accounted for more than 70% of the total effect, suggesting that factors beyond BMI may play a substantial role in the association between night shift frequency and hypertension prevalence. Other pathways, such as circadian rhythm disruption, impaired sleep quality, and neuroendocrine mechanisms involving cortisol dysregulation, may also be involved [26].

These findings suggest that the association between night shift frequency and hypertension prevalence among nursing staff may be influenced by gender, genetic susceptibility, and hospital level. In conclusion, future studies should further investigate the biological and behavioral pathways linking night shift frequency and hypertension prevalence, including circadian rhythm disruption, sleep quality, and neuroendocrine mechanisms.

### Analysis of significant INT_ref_ components between non-shift-related obesity and night shift frequency on hypertension prevalence among female nursing staff, those with parental hypertension, and across hospital levels

This study found that statistically significant INT_ref_ components were observed only among female nursing staff. This finding may be related to women’s greater susceptibility to hormonal fluctuations and circadian rhythm disruption, which may contribute to metabolic abnormalities and increased psychological stress [27]. In addition, blood pressure regulation in women may be more sensitive to weight changes, and obesity may aggravate endothelial dysfunction. As night shift frequency increases, these factors may together be associated with higher hypertension prevalence. Statistically significant INT_ref_ components were observed among nursing staff both with and without parental hypertension, with larger estimates observed among those with parental hypertension. These findings suggest that genetic susceptibility may amplify the interaction between obesity and night shift frequency in relation to hypertension prevalence [28]. It may also indicate that genetic susceptibility and night shift-related occupational exposure jointly contribute to higher hypertension prevalence among nursing staff with parental hypertension [25]. Furthermore, statistically significant INT_ref_ components were identified across different hospital levels, with the largest estimates observed in primary-level hospitals. This finding may be associated with limited medical resources and heavier workloads in primary-level hospitals, which may intensify the combined associations of obesity and night shift frequency with hypertension prevalence.

### Analysis of significant INT_med_ and PIE components among nursing staff in primary-level hospitals with night shift frequencies of “>5 and ≤ 10,” “>10,” and “irregular”

This study found significant INT_med_ and PIE components only among nursing staff in primary-level hospitals with night shift frequencies of “>5 and ≤ 10,” “>10,” and “irregular.” These findings suggest that BMI-related mediated and interaction components may be involved in the association between higher night shift frequency and hypertension prevalence under specific occupational contexts, particularly in primary-level hospitals with relatively high night shift burdens. However, because statistically significant PIE components were not observed in most other subgroups and the CDE accounted for the largest proportion of the total effect, BMI-related mediation appears to be context-dependent rather than a universal mechanism underlying the association between night shift frequency and hypertension prevalence. Consistent with this interpretation, sensitivity analyses modeling BMI as a continuous mediator yielded findings generally consistent with those of the primary analyses using dichotomized BMI. The CDE remained the predominant component in most analyses, whereas BMI-related mediated components were generally limited and were mainly observed among nursing staff working in primary-level hospitals. These findings suggest that the principal interpretation of the four-way decomposition results was not substantially dependent on BMI dichotomization.

BMI-related pathways appeared to be associated with hypertension prevalence primarily among nursing staff in primary-level hospitals when night shifts exceeded five times or were irregular in the past six months. Limited healthcare resources, insufficient recovery opportunities, heavier workloads, and increased psychological stress in primary-level hospitals may contribute to metabolic dysregulation, elevated BMI, and higher hypertension prevalence. Additionally, the tiered healthcare system may impose diverse medical demands on nursing staff, increasing workload and emotional stress, which may contribute to cortisol release and elevated blood pressure [29, 30].

Nursing administrators in primary-level hospitals should optimize night shift scheduling and strengthen health management for nursing staff, particularly among those with frequent or irregular night shifts. Effective resource management and health interventions may help reduce occupational health burdens and support the stable operation of the healthcare system.

### Strengths and limitations

This study used BMI as a potential mediator and applied the four-way decomposition method to analyze the association between night shift frequency and hypertension prevalence among nursing staff. The findings highlighted the complex associations between night shift work and hypertension prevalence. Stratified analyses by gender, parental hypertension status, and hospital level revealed differentiated associations of night shift frequency and obesity with hypertension prevalence in specific groups, providing a basis for personalized interventions.

However, this study also has several limitations. First, this was a cross-sectional study, which limited the ability to establish temporal relationships among night shift frequency, BMI, and hypertension prevalence, and reverse causality could not be excluded. Second, height and weight data were self-reported based on participants’ most recent physical examination results. Although reporting was based on recent physical examination results, reporting bias could not be completely excluded, which may have influenced the estimates of BMI-related mediation and interaction components. Third, although several confounding factors were adjusted for in the analysis, information on dietary intake, sleep duration, medications, work-related factors, and unique hospital identifiers was not available in the original dataset. Consequently, cluster-robust standard errors and multilevel modeling could not be implemented, and residual confounding and potential within-hospital correlation could not be completely excluded. Fourth, night shift frequency was assessed only over the previous six months, and cumulative or long-term night shift exposure was not evaluated. Therefore, the long-term associations between chronic night shift exposure and hypertension prevalence could not be fully assessed. Fifth, while an “irregular” night shift category was included in the analysis, detailed information regarding specific scheduling patterns, rotation characteristics, or the exact number of night shifts within this category was not collected, which may limit the interpretation of the observed associations. Finally, the data were collected between 2016 and 2018, and changes in healthcare work environments, workforce management practices, and occupational health policies over time may affect the interpretation and applicability of the findings. Future research should include broader regional populations and healthcare institutions and incorporate longitudinal designs to better clarify the temporal relationships among night shift frequency, BMI, and hypertension prevalence. Further investigation of the underlying biological mechanisms may provide a more comprehensive basis for occupational health management strategies.

## Conclusion

This study found that higher night shift frequency was associated with higher hypertension prevalence among nursing staff, primarily through direct associations rather than BMI-related pathways. BMI-related interaction components were more prominent among female nurses, those with parental hypertension, and nurses working in primary-level hospitals, while BMI-related mediation components were mainly observed among nursing staff in primary-level hospitals with frequent or irregular night shifts, suggesting that BMI-related mediation may be context-dependent rather than a universal pathway. Nursing administrators and nursing staff should consider these findings when developing scheduling and health management strategies, particularly for nurses with higher BMI, female nurses, those with a family history of hypertension, and those working in primary-level hospitals. These findings also highlight the potential importance of promoting healthier and more sustainable work-life integration through optimized night shift scheduling and occupational health support.

## Supplementary Information

Below is the link to the electronic supplementary material.

Supplementary Material 1

Supplementary Material 2

## Data Availability

The data that support the findings of this study are available from the corresponding author upon reasonable request.

## Abbreviations

BMI: Body Mass Index
SBP: Systolic Blood Pressure
DBP: Diastolic Blood Pressure
CDE: Controlled Direct Effect
INT_ref_: Reference Interaction
INT_med_: Mediated Interaction
PIE: Pure Indirect Effect
