# Supplementary material for: The mediation and interaction of body mass index between night shift frequency and hypertension among nursing staff
Source: BMC Health Serv Res. 2026 Aug 20;26:1580. doi: 10.1186/s12913-026-15399-w (PMC13760765; doi:10.1186/s12913-026-15399-w)
Supplement: Supplementary file 1 [file 12913_2026_15399_MOESM1_ESM.pdf]

Survey Questionnaire on Blood Pressure Status of Nursing Staff

Dear nursing staff,

In order to strengthen the prevention and treatment of hypertension in nursing staff, to analyze the prevalence of hypertension in nursing staff in Hebei province and to explore its influencing factors, we carried out a survey on the status of blood pressure and its related factors in nursing staff in Hebei province. The survey will last about 10 minutes. We will keep all questions that may involve the privacy of you and your family strictly confidential. We sincerely hope that we can get your support and cooperation.

(Note: For blood pressure measurement, both electronic sphygmomanometer and mercury sphygmomanometer were used for 3 consecutive measurements with an interval of 1 to 2 minutes. The mean value of the second and third blood pressure measurements was used as the measurement value, and the first blood pressure measurement was discarded.)

1. **What is your name? [fill in the blank] ***

____

2. **What is your gender？ [Single choice question] ***

A. Female

B. Male

3. **What is your age?[fill in the blank] ***

____

4. **What's the name of your hospital？ (for the determination of hospital level) [fill in the blank] ***

____

5. **Where is your hospital in Hebei Province?[Single choice] ***

A. Shijiazhuang (including Xinji)

B. Baoding (including Dingzhou)

C. Cangju

D. Chengde

E. Handan

F. Hengshui

G. Langfang

H. Qinhuangdao

I. Tangshan

J. Xingtai

K. Zhangjiakou

6. **Which of the following is your department?[Single choice] ***

A. Internal medicine

B. Surgery

C. Obstetrics and Gynecology

D. Pediatrics

E. Emergency Department

F. Operating room
G. Intensive care unit
H. outpatient service
I. Ent Department
J. Administrative logistics
K. Medical technology Department
L. others (Departments in addition to the above departments, such as psychiatry department, radiotherapy and chemotherapy department, rehabilitation department, anesthesiology department, anesthesia recovery room, infection department, general practice department, Traditional Chinese medicine department, intervention department, infectious disease department, integrated traditional Chinese and Western medicine department, dermatology department, medical care center, laser cosmetology department and other departments)

7. What is your degree? [Single choice] *

A. Vocational School Diploma
B. Junior College Diploma
C. Bachelor's Degree
D. Master's Degree
E. Doctorate and Above

8. Which of the following is your current marital status?[Single choice] *

A. Married
B. Unmarried
C. Divorced or Widowed

9. What's your height in centimeters? [Fill in the blanks] *

____

10. What's your weight in kilograms? [Fill in the blanks] *

____

11. What is your systolic blood pressure in mmHg? [Fill in the blanks] *

____

12. What is your diastolic blood pressure in mmHg? [Fill in the blanks] *

____

13. Can your blood pressure be controlled below 140/90mmHg with medications? [Single choice] *

A. Yes
B. No
C. Control targets can be achieved by other means without drugs;
D. No hypertension

14. How many years have you had hypertension now? [Fill in the blanks] *(Note: Less than one year is calculated as one year, and if there is no disease, fill in 0)

____

15. How many years have you had hyperlipidemia now? [Fill in the blanks] *(Note: Less than one year is calculated as one year, and if there is no disease, fill in 0)

____

16. How many years have you had diabetes now? [Fill in the blanks] *(Note: Less than one year is calculated as one year, and if there is no disease, fill in 0)

____

17. Have you taken antihypertensive medication in the last 2 weeks [single choice] *

A. No hypertension

B. Yes

C. No

18. Whether parents had hypertension [single choice] *

A. Yes(At least one parent had hypertension)

B. No(Neither parent had hypertension)

19. How many cigarettes you smoke per day [single choice] *

A. No(No smoking in the past year)

B. less than 10 cigarettes per day

C. 10-20 cigarettes per day

D. more than 20 cigarettes per day

20. How much alcohol do you drink? [Single choice] *

A. None(Not drinking any alcohol in the past 12 months)

B. Occasionally(The average alcohol consumption was less than once per month)

C. Often(The average drinking time was at least 1 time per month)

21. Do you participate in exercise and fitness activities? [Single choice] *(Note: Exercise refers to conscious activities such as running and walking.)

A. Never (not participated in sports for nearly A year)

B. Occasionally (Engage in ≥30min exercise 1-2 times per week)

C. Often (Engage in ≥30min exercise at least 3 times per week)

22. What is your current menstrual status? [Single choice] *

A. Regular menstrual cycle

B. Irregular menstrual cycle

C. Post-menopausal

23. How many times have you been pregnant? [Fill in the blanks] *

____

24. How many times have you given birth? [Fill in the blanks] *

____

25. How many miscarriages have you had? [Fill in the blanks] *

____

26. **Have you experienced pregnancy-induced hypertension? [Single choice] (**Note:Pregnancy-induced hypertensive disorders include gestational hypertension, preeclampsia, eclampsia, chronic hypertension with superimposed preeclampsia, and chronic hypertension during pregnancy. This question specifically refers to gestational hypertension, preeclampsia, and eclampsia, defined as blood pressure ≥140/90 mmHg first occurring during pregnancy and returning to normal within 12 weeks postpartum; with proteinuria (-) or (+), (++); or seizures occurring on

this basis.)

A. Yes

B. No

C. No history of pregnancy

27. Do you have a history of oral contraceptives? [single choice] *

A. Yes

B. No

28. Have you received hormone replacement therapy? [Single choice] *(Note: Hormone replacement therapy refers to the treatment of postmenopausal women with estrogen deficiency by supplementing estrogen and progestin to relieve their menopausal symptoms.)

A. Yes

B. No

29. What is your average number of night shifts per month in recent half a year? [Fill in the blanks] *

____

30. Do you have any other medical history? **[Fill in the blanks] *(Note:If not, fill in 0)**

____
