# Supplementary material for: The mediation and interaction of body mass index between night shift frequency and hypertension among nursing staff
Source: BMC Health Serv Res. 2026 Aug 20;26:1580. doi: 10.1186/s12913-026-15399-w (PMC13760765; doi:10.1186/s12913-026-15399-w)
Supplement: Supplementary file 2 [file 12913_2026_15399_MOESM2_ESM.docx]

| Table S1. Sensitivity Analysis of the Overall Four-Way Decomposition of the Association Between Night Shift Frequency and Hypertension Prevalence Among Nursing Staff, with BMI Modeled as a Continuous Mediator | | | | |
| --- | --- | --- | --- | --- |
|  | Continuous BMI as the Mediator [Estimate (95% CI)] | | | |
| Night Shift Frequency Grouping in the Past Six Months | >0 and ≤5 | ＞5 and ≤10 | ＞10 | Irregular |
| Total effect | 1.09 (1.06, 1.11)* | 1.18 (1.13, 1.23)* | 1.28 (1.20, 1.37)* | 1.39 (1.27, 1.51)* |
| ERR due to each component |  |  |  |  |
| CDE | 0.07 (0.05, 0.09)* | 0.15 (0.10, 0.20)* | 0.24 (0.16, 0.31)* | 0.33 (0.22, 0.44)* |
| INT_ref_ | 0.02 (0.01, 0.03)* | 0.04 (0.02, 0.06)* | 0.06 (0.03, 0.10)* | 0.09 (0.05, 0.14)* |
| INT_med_ | -0.00 (-0.00, -0.00)* | -0.00 (-0.00, -0.00)* | -0.00 (-0.01, -0.00)* | -0.01 (-0.02, -0.00)* |
| PIE | -0.00 (-0.01, -0.00)* | -0.01 (-0.01, -0.00)* | -0.01 (-0.02, -0.00)* | -0.01 (-0.03, -0.00)* |
| Proportion of effect due to each component |  |  |  |  |
| CDE | 0.83 (0.73, 0.93)* | 0.83 (0.73, 0.93)* | 0.83 (0.72, 0.93)* | 0.83 (0.72, 0.93)* |
| INT_ref_ | 0.22 (0.12, 0.32)* | 0.22 (0.12, 0.33)* | 0.23 (0.12, 0.33)* | 0.23 (0.13, 0.34)* |
| INT_med_ | -0.00 (-0.01, -0.00)* | -0.01 (-0.02, -0.00)* | -0.01 (-0.03, -0.00)* | -0.02 (-0.04, -0.00)* |
| PIE | -0.04 (-0.08, -0.01)* | -0.04 (-0.08, -0.01)* | -0.04 (-0.08, -0.01)* | -0.04 (-0.07, -0.01)* |
| Note: **P*<0.05. BMI:Body Mass Index; CDE:controlled direct effect; INT_ref_:reference interaction; INT_med_:mediated interaction; PIE:pure indirect effect. | | | | |

|  | Table S2. Sensitivity Analysis of the Four-Way Decomposition of the Association Between Night Shift Frequency and Hypertension Prevalence Among Female and Male Nursing Staff, with BMI Modeled as a Continuous Mediator | | | | | | |
| --- | --- | --- | --- | --- | --- | --- | --- |
|  |  | Continuous BMI as the Mediator [Estimate (95% CI)] | | | | | |
| Night Shift Frequency Grouping in the Past Six Months | Group | >0 and ≤5 | ＞5 and ≤10 | | | ＞10 | Irregular |
| Total effect | Female | 1.06 (1.03, 1.08)* | 1.12 (1.06, 1.17)* | | | 1.18 (1.10, 1.27)* | 1.25 (1.13, 1.38)* |
|  | Male | 1.11 (1.03, 1.18)* | 1.23 (1.06, 1.40)* | | | 1.35 (1.09, 1.66)* | 1.50 (1.12, 1.97)* |
| Total effect  ERR due to each component |  |  |  | | |  |  |
| CDE | Female | 0.06 (0.03, 0.08)* | 0.11 (0.06, 0.16)* | | | 0.18 (0.10, 0.26)* | 0.24 (0.13, 0.36)* |
|  | Male | 0.07 (0.00, 0.14)* | 0.14 (0.00, 0.29)* | | | 0.22 (0.01, 0.47)* | 0.30 (0.01, 0.67)* |
| INT_ref_ | Female | 0.01 (0.00, 0.02)* | 0.02 (0.00, 0.04)* | | | 0.03 (0.00, 0.07)* | 0.04 (0.00, 0.09)* |
|  | Male | 0.02 (-0.03, 0.07) | 0.04 (-0.06, 0.14) | | | 0.07 (-0.08, 0.23) | 0.10 (-0.10, 0.34) |
| INT_med_ | Female | -0.00 (-0.00, -0.00)* | -0.00 (-0.00, -0.00)* | | | -0.00 (-0.01, -0.00)* | -0.01 (-0.02, -0.00)* |
|  | Male | 0.00 (-0.00, 0.00) | 0.00 (-0.01, 0.02) | | | 0.01 (-0.03, 0.05) | 0.02 (-0.05, 0.09) |
| PIE | Female | -0.01 (-0.01, -0.00)* | -0.01 (-0.02, -0.01)* | | | -0.02 (-0.03, -0.01)* | -0.03 (-0.04, -0.02)* |
|  | Male | 0.02 (0.00, 0.03)* | 0.04 (0.01, 0.07)* | | | 0.06 (0.01, 0.10)* | 0.07 (0.02, 0.14)* |
| Proportion of effect due to each component |  | | |  |  | |  |
| CDE | Female | 0.95 (0.80, 1.17)* | 0.96 (0.80, 1.17)* | | | 0.96 (0.80, 1.16)* | 0.96 (0.80, 1.15)* |
|  | Male | 0.64 (0.05, 1.17)* | 0.63 (0.05, 1.20)* | | | 0.62 (0.05, 1.22)* | 0.61 (0.04, 1.25)* |
| INT_ref_ | Female | 0.17 (0.01, 0.35)* | 0.17 (0.01, 0.36)* | | | 0.17 (0.01, 0.37)* | 0.18 (0.01, 0.38)* |
|  | Male | 0.18 (-0.47, 0.67) | 0.19 (-0.46, 0.64) | | | 0.20 (-0.44, 0.62) | 0.21 (-0.41, 0.60) |
| INT_med_ | Female | -0.01 (-0.02, -0.00)* | -0.01 (-0.03, -0.00)* | | | -0.02 (-0.05, -0.00)* | -0.03 (-0.06, -0.01)* |
|  | Male | 0.01 (-0.03, 0.05) | 0.02 (-0.07, 0.09) | | | 0.03 (-0.12, 0.13) | 0.04 (-0.17, 0.17) |
| PIE | Female | -0.12 (-0.25, -0.06)* | -0.11 (-0.24, -0.06)* | | | -0.11 (-0.23, -0.06)* | -0.11 (-0.23, -0.06)* |
|  | Male | 0.17 (0.04, 0.55)* | 0.16 (0.04, 0.55)* | | | 0.16 (0.03, 0.55)* | 0.15 (0.03, 0.55)* |
| Note: **P*<0.05. BMI:Body Mass Index; CDE:controlled direct effect; INT_ref_:reference interaction; INT_med_:mediated interaction; PIE:pure indirect effect. | | | | | | | |

|  | Table S3. Sensitivity Analysis of the Four-Way Decomposition of the Association Between Night Shift Frequency and Hypertension Prevalence Among Nursing Staff With and Without Parental Hypertension, With BMI Modeled as a Continuous Mediator | | | | | |
| --- | --- | --- | --- | --- | --- | --- |
|  | |  | Continuous BMI as the Mediator [Estimate (95% CI)] | | | |
| Night Shift Frequency Grouping in the Past Six Months | | Group | >0 and ≤5 | ＞5 and ≤10 | ＞10 | Irregular |
| Total effect | | Parents with Hypertension | 1.11 (1.07, 1.14)* | 1.23 (1.15, 1.30)* | 1.36 (1.24, 1.49)* | 1.50 (1.33, 1.69)* |
|  | | Parents without Hypertension | 1.07 (1.03, 1.10)* | 1.14 (1.07, 1.22)* | 1.21 (1.10, 1.34)* | 1.29 (1.14, 1.48)* |
| Total effect  ERR due to each component | |  |  |  |  |  |
| CDE | | Parents with Hypertension | 0.09 (0.06, 0.12)* | 0.18 (0.12, 0.25)* | 0.28 (0.18, 0.40)* | 0.40 (0.25, 0.56)* |
|  | | Parents without Hypertension | 0.06 (0.03, 0.09)* | 0.12 (0.05, 0.19)* | 0.18 (0.08, 0.30)* | 0.25 (0.11, 0.42)* |
| INT_ref_ | | Parents with Hypertension | 0.02 (0.01, 0.04)* | 0.05 (0.02, 0.08)* | 0.08 (0.04, 0.13)* | 0.12 (0.05, 0.19)* |
|  | | Parents without Hypertension | 0.01 (0.00, 0.03)* | 0.03 (0.00, 0.05)* | 0.05 (0.00, 0.09)* | 0.06 (0.00, 0.13)* |
| INT_med_ | | Parents with Hypertension | -0.00 (-0.00, 0.00) | -0.00 (-0.00, 0.00) | -0.00 (-0.01, 0.00) | -0.01 (-0.02, 0.00) |
|  | | Parents without Hypertension | -0.00 (-0.00, -0.00)* | -0.00 (-0.00, -0.00)* | -0.00 (-0.01, -0.00)* | -0.01 (-0.02, -0.00)* |
| PIE | | Parents with Hypertension | -0.00 (-0.01, 0.00) | -0.01 (-0.01, 0.00) | -0.01 (-0.02, 0.00) | -0.01 (-0.03, 0.00) |
|  | | Parents without Hypertension | -0.00 (-0.01, -0.00)* | -0.01 (-0.01, -0.00)* | -0.01 (-0.02, -0.00)* | -0.02 (-0.03, -0.00)* |
| Proportion of effect due to each component | |  |  |  |  |  |
| CDE | | Parents with Hypertension | 0.81 (0.69, 0.93)* | 0.80 (0.68, 0.93)* | 0.80 (0.68, 0.93)* | 0.80 (0.67, 0.92)* |
|  | | Parents without Hypertension | 0.86 (0.67, 1.07)* | 0.86 (0.67, 1.07)* | 0.86 (0.67, 1.07)* | 0.86 (0.66, 1.06)* |
| INT_ref_ | | Parents with Hypertension | 0.23 (0.11, 0.36)* | 0.23 (0.11, 0.37)* | 0.24 (0.11, 0.38)* | 0.24 (0.12, 0.38)* |
|  | | Parents without Hypertension | 0.21 (0.01, 0.44)* | 0.21 (0.02, 0.45)* | 0.21 (0.02, 0.47)* | 0.22 (0.02, 0.48)* |
| INT_med_ | | Parents with Hypertension | -0.00 (-0.01, 0.00) | -0.01 (-0.02, 0.00) | -0.01 (-0.03, 0.00) | -0.02 (-0.04, 0.00) |
|  | | Parents without Hypertension | -0.01 (-0.01, -0.00)* | -0.01 (-0.03, -0.00)* | -0.02 (-0.05, -0.00)* | -0.02 (-0.06, -0.00)* |
| PIE | | Parents with Hypertension | -0.03 (-0.08, 0.01) | -0.03 (-0.07, 0.01) | -0.03 (-0.07, 0.01) | -0.02 (-0.07, 0.01) |
|  | | Parents without Hypertension | -0.06 (-0.16, -0.01)* | -0.06 (-0.16, -0.01)* | -0.06 (-0.16, -0.01)* | -0.05 (-0.15, -0.01)* |
| Note: **P*<0.05. BMI:Body Mass Index; CDE:controlled direct effect; INT_ref_:reference interaction; INT_med_:mediated interaction; PIE:pure indirect effect. | | | | | | |

|  | Table S4. Sensitivity Analysis of the Four-Way Decomposition of the Association Between Night Shift Frequency and Hypertension Prevalence Among Nursing Staff in Hospitals of Different Levels, With BMI Modeled as a Continuous Mediator | | | | | |
| --- | --- | --- | --- | --- | --- | --- |
|  | |  | Continuous BMI as the Mediator [Estimate (95% CI)] | | | |
| Night Shift Frequency Grouping in the Past Six Months | | Group | >0 and ≤5 | ＞5 and ≤10 | ＞10 | Irregular |
| Total effect | | Primary-level | 1.26 (1.15, 1.38)* | 1.60 (1.33, 1.91)* | 2.04 (1.56, 2.64)* | 2.60 (1.82, 3.63)* |
|  | | Secondary | 1.08 (1.05, 1.12)* | 1.17 (1.10, 1.25)* | 1.27 (1.15, 1.39)* | 1.37 (1.21, 1.55)* |
|  | | Tertiary | 1.07 (1.03, 1.11)* | 1.15 (1.07, 1.23)* | 1.23 (1.10, 1.37)* | 1.32 (1.14, 1.51)* |
| Total effect  ERR due to each component | |  |  |  |  |  |
| CDE | | Primary-level | 0.16 (0.06, 0.26)* | 0.34 (0.12, 0.58)* | 0.55 (0.18, 0.98)* | 0.79 (0.25, 1.46)* |
|  | | Secondary | 0.08 (0.04, 0.11)* | 0.16 (0.09, 0.23)* | 0.24 (0.14, 0.36)* | 0.34 (0.19, 0.50)* |
|  | | Tertiary | 0.06 (0.03, 0.10)* | 0.13 (0.05, 0.20)* | 0.19 (0.08, 0.32)* | 0.27 (0.11, 0.44)* |
| INT_ref_ | | Primary-level | 0.07 (0.04, 0.12)* | 0.18 (0.10, 0.29)* | 0.32 (0.16, 0.54)* | 0.49 (0.24, 0.86)* |
|  | | Secondary | 0.01 (-0.00, 0.03) | 0.03 (-0.00, 0.06) | 0.04 (-0.00, 0.09) | 0.06 (-0.00, 0.13) |
|  | | Tertiary | 0.02 (0.01, 0.03)* | 0.04 (0.01, 0.07)* | 0.06 (0.02, 0.11)* | 0.09 (0.02, 0.16)* |
| INT_med_ | | Primary-level | 0.01 (0.00, 0.02)* | 0.05 (0.02, 0.08)* | 0.12 (0.05, 0.23)* | 0.25 (0.10, 0.50)* |
|  | | Secondary | -0.00 (-0.00, -0.00)* | -0.00 (-0.00, -0.00)* | -0.00 (-0.01, -0.00)* | -0.01 (-0.02, -0.00)* |
|  | | Tertiary | -0.00 (-0.00, -0.00)* | -0.00 (-0.01, -0.00)* | -0.01 (-0.01, -0.00)* | -0.01 (-0.03, -0.00)* |
| PIE | | Primary-level | 0.02 (0.01, 0.03)* | 0.04 (0.02, 0.06)* | 0.05 (0.03, 0.09)* | 0.07 (0.03, 0.13)* |
|  | | Secondary | -0.01 (-0.01, -0.00)* | -0.01 (-0.02, -0.00)* | -0.02 (-0.03, -0.01)* | -0.02 (-0.04, -0.01)* |
|  | | Tertiary | -0.01 (-0.01, -0.00)* | -0.01 (-0.02, -0.00)* | -0.02 (-0.03, -0.00)* | -0.02 (-0.04, -0.01)* |
| Proportion of effect due to each component | |  |  |  |  |  |
| CDE | | Primary-level | 0.61 (0.37, 0.73)* | 0.57 (0.32, 0.71)* | 0.53 (0.28, 0.68)* | 0.49 (0.25, 0.66)* |
|  | | Secondary | 0.92 (0.76, 1.13)* | 0.92 (0.76, 1.12)* | 0.92 (0.76, 1.11)* | 0.92 (0.76, 1.10)* |
|  | | Tertiary | 0.83 (0.62, 1.02)* | 0.83 (0.62, 1.02)* | 0.83 (0.61, 1.02)* | 0.83 (0.61, 1.02)* |
| INT_ref_ | | Primary-level | 0.29 (0.16, 0.49)* | 0.30 (0.17, 0.50)* | 0.30 (0.17, 0.50)* | 0.31 (0.18, 0.50)* |
|  | | Secondary | 0.15 (-0.02, 0.34) | 0.16 (-0.02, 0.35) | 0.16 (-0.01, 0.36) | 0.16 (-0.01, 0.37) |
|  | | Tertiary | 0.25 (0.08, 0.53)* | 0.26 (0.08, 0.54)* | 0.26 (0.08, 0.56)* | 0.27 (0.08, 0.58)* |
| INT_med_ | | Primary-level | 0.04 (0.02, 0.07)* | 0.08 (0.04, 0.13)* | 0.12 (0.06, 0.19)* | 0.16 (0.08, 0.25)* |
|  | | Secondary | -0.01 (-0.01, -0.00)* | -0.01 (-0.03, -0.00)* | -0.02 (-0.04, -0.00)* | -0.02 (-0.05, -0.00)* |
|  | | Tertiary | -0.01 (-0.02, -0.00)* | -0.02 (-0.04, -0.00)* | -0.03 (-0.07, -0.00)* | -0.03 (-0.09, -0.01)* |
| PIE | | Primary-level | 0.07 (0.03, 0.14)* | 0.06 (0.03, 0.12)* | 0.05 (0.02, 0.11)* | 0.05 (0.02, 0.10)* |
|  | | Secondary | -0.07 (-0.16, -0.02)* | -0.07 (-0.16, -0.02)* | -0.06 (-0.15, -0.02)* | -0.06 (-0.14, -0.02)* |
|  | | Tertiary | -0.07 (-0.21, -0.02)* | -0.07 (-0.20, -0.02)* | -0.07 (-0.20, -0.02)* | -0.07 (-0.19, -0.02)* |
| Note: **P*<0.05. BMI:Body Mass Index; CDE:controlled direct effect; INT_ref_:reference interaction; INT_med_:mediated interaction; PIE:pure indirect effect. | | | | | | |
